# Lignin-Based Polyurethanes: Opportunities for Bio-Based Foams, Elastomers, Coatings and Adhesives

**DOI:** 10.3390/polym11071202

**Published:** 2019-07-18

**Authors:** Mona Alinejad, Christián Henry, Saeid Nikafshar, Akash Gondaliya, Sajad Bagheri, Nusheng Chen, Sandip K. Singh, David B. Hodge, Mojgan Nejad

**Affiliations:** 1 Department of Forestry, Michigan State University, East Lansing, MI 48824, USA; 2 Chemical Engineering and Materials Science, Michigan State University, East Lansing, MI 48824, USA; 3 Eastern Regional Research Center, USDA-ARS, Wyndmoor, PA 19038, USA; 4 Chemical & Biological Engineering, Montana State University, Bozeman, MT 59717, USA; 5 Biochemical Process Engineering, Division of Chemical Engineering, Department of Civil, Environmental and Natural Resources Engineering, Luleå University of Technology, SE-971 87 Luleå, Sweden

**Keywords:** lignin, polyurethane, adhesives, foams, coatings, elastomers

## Abstract

Polyurethane chemistry can yield diverse sets of polymeric materials exhibiting a wide range of properties for various applications and market segments. Utilizing lignin as a polyol presents an opportunity to incorporate a currently underutilized renewable aromatic polymer into these products. In this work, we will review the current state of technology for utilizing lignin as a polyol replacement in different polyurethane products. This will include a discussion of lignin structure, diversity, and modification during chemical pulping and cellulosic biofuels processes, approaches for lignin extraction, recovery, fractionation, and modification/functionalization. We will discuss the potential of incorporation of lignins into polyurethane products that include rigid and flexible foams, adhesives, coatings, and elastomers. Finally, we will discuss challenges in incorporating lignin in polyurethane formulations, potential solutions and approaches that have been taken to resolve those issues.

## 1. Introduction

### 1.1. Polyurethanes

Polyurethanes (PUs) are a diverse class of polymers representing nearly 6% of the total global polymer market with applications that include coatings, adhesives, foams, elastomers, and fibers [1]. These products are used in diverse market segments that include packaging, building and construction, automotive, electronics, and biomedical products among others. This diversity in polymer products, markets, and applications is due to the enormous possibilities for defining chemical composition and polymer structure during synthesis which, in turn, can impart polymer products with diverse possibilities for physicochemical properties [2,3].

The common feature of all polyurethanes is the urethane linkage (–NH–(C=O)–O–), which is a carbamate ester linkage synthesized by reaction between an isocyanate (–NCO) group and an alcohol (–OH). For the synthesis of polymers and crosslinked polymer networks, one monomer must contain at least two isocyanates (i.e., a diisocyanate) and the other monomer must contain at least two alcohols (i.e., a diol or a polyol). Diisocyanates can be aromatic or aliphatic and, generally, aromatic isocyanate such as methylene diphenyl diisocyanate (MDI) and toluene diisocyanate (TDI) have a higher reactivity compared with the aliphatic isocyanates such as hexamethylene diisocyanate (HMDI). Recently, bio-based diisocyanates such as penta-methylene diisocyanate (PDI) have been commercialized to enable 100% bio-based polyurethanes [4]. The structure and properties of the polyurethane product depend on the type of the diisocyanate, the polyol, and the synthesis process [2]. 

Reaction of a diisocyanate with a diol will result in a linear polymer, while utilization of polyol with three or more hydroxyl groups will result in crosslinked polymer networks. A diverse range of polyols are currently used in polyurethane applications, with common polyols including low molecular weight diols (e.g., propylene glycol, ethylene glycol, 1,4 butanediol) and low molecular weight polymers (i.e., <10 kDa) generally containing 2-8 hydroxyl groups per mole [5]. Commonly used polymeric polyols include polyethers typically derived from polymerization of alkylene oxides such as propylene oxide and a diverse range of aliphatic and aromatic polyester polyols. Bio-based polyols are a rapidly growing source of polyols and currently include compounds derived from triglycerides and sugars such as glucose, sucrose, sorbitol, and glycerol that can be used as initiators for synthesis of polyether polyols [5]. Globally, polyurethanes had a market size of $60.5 billion in 2017 corresponding to 16.9 million tonnes of product and have seen continuous growth in recent years with annual growth rates of 8%–10% that are expected to continue into at least the near-term future [6]. This growth is driven by increased demand from the end market segments and substantial growth in emerging markets (e.g., China, India, and Brazil). “Green polyols” that include bio-based polyols and polycarbonate polyols that incorporate CO_2_ represented approximately 13% of the $19 billion polyol market in 2015 [7]. The global polyol market is expected to exhibit the same strong growth as the polyurethane market, while the share of green polyols is expected to increase indicating that bio-based polyols will represent an enormous opportunity for new growth.

### 1.2. Opportunities and Challenges for Lignin Incorporation into Polyurethanes

The diverse possibilities for chemical compositions provide an opportunity for the integration of novel, bio-based feedstocks into polyurethane products. The total or partial replacement of fossil-derived polymer precursors (i.e., isocyanates, polyols, and other additives) with renewable, bio-based feedstocks is one route to improve the sustainability of these products [8]. Lignins are a class of aromatic polymers that comprise one of the major components of the cell walls of plants and represent a large potential resource for renewable polymers. Lignins derived from chemical pulping processes and biorefining processes are promising candidates for use in polyurethane synthesis (generally as a polyol) if well-established challenges can be overcome. In addition to increasing the bio-based content of the polyurethane products, lignin incorporation into various polyurethane products has been shown in some cases to provide performance advantages that include enhanced crosslinking density, improved biodegradability, increased ultraviolet (UV) stability, antioxidant properties, and improved mechanical strength and thermal stability of the final product [9,10]. 

Several challenges have been identified for the replacement of conventional polyols with lignins that have limited its utility in polyurethanes with a summary of these challenges outlined in Table 1. First, the reactivity of hydroxyl groups within lignin towards an isocyanate may be restricted due to the steric hindrance as a consequence of both the higher order structure [11] and, potentially, self-association of the lignin polymer that limits access to hydroxyl groups. Furthermore, alcohols within a lignin polymer have different intrinsic reactivities with an isocyanate group depending on whether the alcohol is a primary (1°), secondary (2°), or phenolic (Ph) hydroxyl group. The relative reactivities of these alcohols with an isocyanate group as determined by uncatalyzed reaction rate can be ranked as 1° > 2° ≫ Ph with a phenolic hydroxyl group exhibiting up to a 1000-fold lower rate of reaction relative to a primary alcohol [12]. 

Lignins must be soluble in the solvent used for reaction or with other polyols used in the synthesis, which may be a challenge for some applications. Lignins derived from the alkaline delignification of grasses, hardwoods, and softwoods are partially or completely soluble in many organic solvents [13], with many lignins exhibiting the general trend of increasing solubility with increasing solvent polarity in organic solvents [14]. Furthermore, polydisperse lignins can exhibit differing solubilities and reactivities [15] that result in reaction of only a subset of the original lignin sample.

Many types of lignin may contain either sulfur such as kraft lignins, lignosulfonates, and lignins subjected to pretreatments that include sulfur such as dilute acid and some ionic liquid pretreatments. This restricts their utility in some polymer applications as sulfur can contribute to odor problems in final products while product yellowing due to sulfur can be problematic for coating applications. Removing sulfur, particularly covalently bound sulfur may not be an economically feasible solution. Therefore, sulfur-free lignins (including many potential biorefinery-derived lignins), organosolv or low-sulfur kraft lignins may be more desirable for polyurethane reactions [15]. In addition, the dark color of many lignins limit their application in coatings as the transparency would decrease dramatically [15]. However, lignin-based resins can be used for formulating dark color stains for wood products, formulating dark color primers for architectural coatings, or in industrial coatings where the product performance is more important than the color.

UV instability is another problem for many lignin-containing polyurethane products that contain unreacted phenolic hydroxyl groups. Additionally, lignin is primary contributor to the discoloration of wood in exterior applications [16]. There is no published work on the photostability of lignin-based polyurethane coatings in the presence of UV stabilizers although there are some studies that have applied lignin nanoparticles as UV stabilizer agents [17,18,19,20,21]. At high loadings, lignin was demonstrated to contribute to the photodegradation process. However, one study demonstrated that adding aminated lignin as a reagent can improve the photostability of the final lignin-based coatings [22]. 

Another challenge for the utilization of lignin in polyurethane applications is its high values for *T_g_*, with values typically ranging from 90–150 °C [23], while a solvent-fractionated commercial softwood lignin (Indulin AT) yielded fractions with values of *T_g_* ranging from 77 to 173 °C [14]. As a result of this, many lignins are limited to flexible polymers such as polyurethane coatings since these require high tensile strength and elongation at break and loading high contents of lignins increase the brittleness of the resulting PUs [24]. Approaches to decreasing the *T_g_* of lignin-based PU samples to make the coating more flexible include modification of the lignin through, for example, depolymerization or functionalization. 

Importantly, most of these challenges have been identified for unfractionated, unfunctionalized kraft lignins. Consequently, as outlined in Table 1, lignins from other sources and other processing approaches (e.g., biorefining processes), subjected to different purification, recovery, or fractionation approaches, and potentially subjected to chemical modifications such as functionalization or depolymerization represent potential pathways for integrating novel lignins into polyurethane products.

## 2. Impact of Source, Processing History, and Functionalization on Lignin Structure 

### 2.1. Diversity of Native Lignins

**Figure 1:**
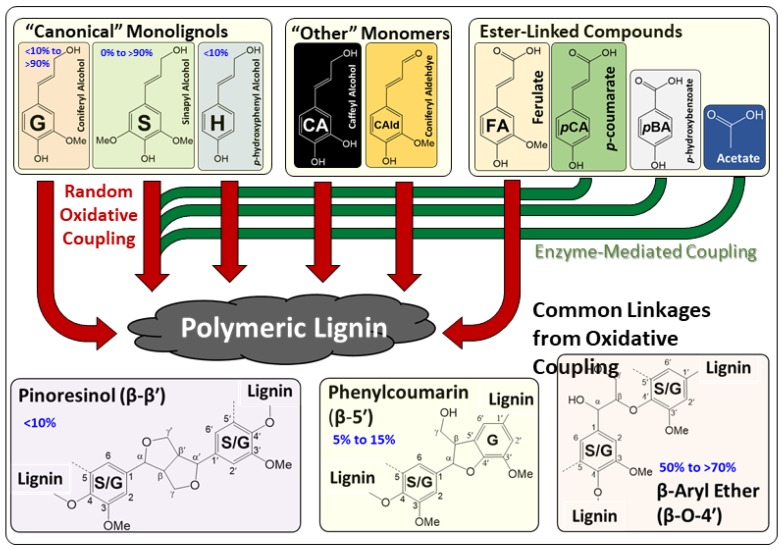
Key compositional and structural features of lignins arising from lignin biosynthesis.

Lignin is the most abundant renewable aromatic polymer and represents an enormous reservoir of renewable carbon with the potential to serve as a feedstock for fuels, chemicals, and materials. Together with the polysaccharides cellulose and hemicellulose, lignin is one of the principal structural polymers of the cell walls of terrestrial plants and can comprise up to 30% of the mass of a plant on a dry basis. The composition and properties of the lignin in a plant prior to processing are a complex function of its source derived from plant taxonomy and genotype, development stage, environment, and tissue type [25]. Gymnosperm lignins are comprised primarily of monomers derived from coniferyl alcohol, corresponding to the guaiacyl or “G” monomer upon incorporation into the lignin polymer, while angiosperm lignins are comprised primarily of monomers derived from coniferyl and sinapyl alcohols (syringyl or “S” monomer) (Figure 1) [26]. Additionally, lignins can contain minor amounts (<5%) of monomers derived from *p*-coumaryl alcohol (*p*-hydroxyphenyl or “H” monomer) as well as a number of “non-canonical” monomers and esters substitutions involving *p*-coumarate (*p*CA), ferulate (FA), *p*-hydroxybenzoate (*p*BA), and acetate [27]. These monomers are incorporated into the growing lignin polymer via oxidative coupling and result in a random distribution of monomers and linkages, with the majority of these linkages comprised of β-O-4′ linkages, β-5′ linkages, and β-β′ linkages when the conventional S and G monomers are utilized (Figure 1). Ferulate and di-ferulates are incorporated into hemicelluloses and can participate in oxidative coupling reactions with lignin [28]. Other carboxylic acids including *p*-coumarate, *p*-hydroxybenzoate, and acetate can incorporated into lignin by enzyme-mediated acylation, presumably with monolignols prior to incorporation into the lignin polymer [29]. 

### 2.2. Industrial Approaches to Yield Process-Modified Lignins

Lignins that would be considered as a source of renewable materials or chemicals are significantly modified from the native lignins within the plant cell wall. The properties of these process-modified lignins are a function of the conditions used during processing and subsequent steps that may include extraction, recovery, purification, fractionation, and/or functionalization/derivatization. Chemical pulping processes currently represent the largest existing source for process-modified lignins. These lignins are derived from processes that solubilize lignin from the cell wall by alkaline delignification using alkali and Na_2_S (kraft pulping), alkali (soda pulping), or by lignin sulfonation (sulfite pulping processes) [26].

Applications beyond combustion for lignins recovered from chemical pulping liquors have been the subject of more than a century of research and development with large markets currently developed for lignosulfonates derived from sulfite pulping processes. These include commercial applications for lignosulfonates as a water reducer for concrete, dispersant, surfactants, as a chelator, and as a feedstock for the production of vanillin [26]. While lignosulfonates represent approximately 5% of the 70 million tonnes per year of lignin generated from chemical pulping processes [30] these comprise >90% of the commercial lignin products [31]. Major structural changes during chemical pulping processes include partial depolymerization and sulfonation of lignins during sulfite pulping processes while alkaline and kraft pulping processes result in significant depolymerization of lignin by cleavage of ether bonds and repolymerization of these fragments to form new C–C bonds through condensation reactions [32]. These modifications, including this repolymerization, significantly limit the utility of kraft and alkali lignins for many applications. As one example, a phenolic hydroxyl group is formed when a β-aryl ether is cleaved during alkaline delignification that, as discussed earlier, has a substantially lower reactivity with an isocyanate relative to a primary or secondary alcohol.

Another promising source of lignins includes biorefining processes that utilize the polysaccharides (and potentially the lignins as well) within the cell walls of plants for the production of renewable fuels, chemicals, and polymeric materials [33]. A diverse range of process chemistries, often in combination with polysaccharide hydrolysis with cellulolytic enzymes, have been proposed and employed for “deconstructing” cell wall biopolymers into monomeric sugars and potentially lignins and lignin-derived aromatics. These processes span the diverse spectrum of pH (acidic to alkaline) and can employ aqueous or organic solvents that facilitate solubilization and/or fractionation of classes of cell wall biopolymers [34]. The lignins derived from these processes offer enormous potential for both adding value to the biorefining processes and can offer a diverse range of properties that have not been as thoroughly explored in potential product applications as the well-studied lignins derived from conventional chemical pulping processes. As one example, lignins derived from non-wood feedstocks (e.g., corn stover, switchgrass, rice straw, sugarcane bagasse, etc.) exhibit substantial structural differences relative to woody feedstocks [28]. These feedstocks represent a small fraction of the global chemical pulping market but may represent a significant feedstock for biorefining processes [35]. As another example, biorefinery-derived lignins can be subjected to significantly different processing chemistries from conventional chemical pulping processes and, consequently, can exhibit significantly different properties with important implications for the suitability of these lignins in co-products applications such as polyurethanes.

**Figure 2:**
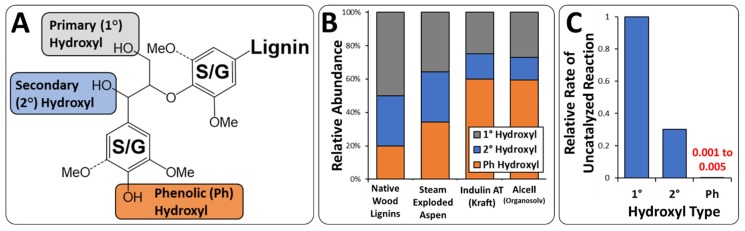
Identification of different pools of hydroxyl groups (**A**), their alteration (**B**) during processing based on [36,37] and (**C**) relative rate of reaction of an alcohol with an isocyanate based on [12].

For polyurethane applications, the type, abundance, and accessibility of hydroxyl groups in the lignin is critical. Importantly, the relative abundance and distribution of these alcohol groups is strongly dependent on the biomass source and processing history. It has been demonstrated that “native” hardwood and softwood milled-wood lignins can have 1°:2°:Ph ratios of approximately 5:3:2 as determined by ^31^P nuclear magnetic resonance (NMR) [36,37]. Process-modified lignins may have a much higher proportion of phenolic hydroxyl groups as these groups are formed upon lignin depolymerization. Illustrative examples include increases in phenolic hydroxyl content as determined by ^31^P NMR in aspen lignin following steam explosion pretreatment, a commercially available softwood kraft lignin (Indulin AT), and an acidic ethanol organosolv pretreatment of mixed hardwoods (Alcell lignin) (Figure 2B). For all these lignins, the total hydroxyl content was in the range of approximately 5–7 mmol OH per g lignin [37]. 

### 2.3. Lignin Fractionation

“Lignin” is not a homogeneous, monodisperse polymer, but rather a complex mixture of polymers that may exhibit a diverse range of molar masses (e.g., polydispersity). Significant differences in structures between polymers (monomer abundance, interunit linkages), and potential diverse distributions of various functional groups (e.g., primary, secondary, and phenolic hydroxyl and carboxylate groups) between polymer chains also contribute to differences in the bulk properties of lignin (e.g., solubility, reactivity with an isocyanate). As described above, these features arise from a lignin’s biological source and its processing history. Besides the role of biomass source and processing history in determining the structure and properties of the lignin, lignin fractionation based on differences in physical properties also provides a route to yield process lignins with properties that are tailored for a target application such as incorporation into polyurethane products [38]. 

**Figure 3:**
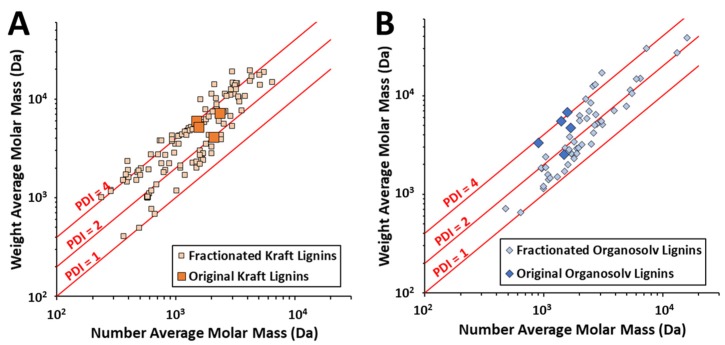
Demonstration of lignin fractionation to yield diverse molar masses from (**A**) Kraft lignins from [39,40,41,42,43,44,45] and (**B**) organosolv lignins from [46,47,48,49,50]. Contours for polydispersity index (PDI) are presented for reference.

Lignin fractionation can be performed using approaches that selectively recover or separate a subset of lignins enriched or depleted in certain properties (e.g., molar mass, aromatic hydroxyl content, hydrophilicity, etc.) using solubility-based fractionation (e.g., selective solubilization or precipitation of process-solubilized lignins) [14] or membrane separations (e.g., ultrafiltration of process-solubilized lignins). As an example, a number of studies employing solvent fractionation to yield subsets of lignins that are more monodisperse at different molar masses are presented in Figure 3. It should be considered that any process to further fractionate, modify, or functionalize the lignin will add cost to the lignin, which is already a low-value feedstock, and may render subsequent utilization economically prohibitive. 

### 2.4. Lignin Functionalization 

Aliphatic and phenolic hydroxyl groups within lignins (Figure 2A) are the key structural features that allow lignin to be utilized as a polyol macromonomer in polyurethanes [51]. However, other features of lignins such as molar mass, polydispersity, solubility in the reaction solvent, and impurities (e.g., inorganics and polysaccharides) impact both the reactivity of a lignin hydroxyl group with an isocyanate and the resulting properties of the final products [9,52,53,54,55]. While lignin can be functionalized to contain isocyanate groups [56], significant work has been directed at its utilization as a polyol macromonomer for application in polyurethanes. General strategies for incorporating lignin as a polyol macromonomer into polyurethanes include utilizing the lignin or lignin fraction “as is” either alone or with other polyols or by first functionalizing the lignin to increase either the hydroxyl content, increase the aliphatic hydroxyl content, or increase the chemical accessibility of aliphatic hydroxyl groups [52]. While functionalization provides the opportunity to yield significant improvements in the processability of the lignin, utilizing unmodified lignins in polyurethane synthesis would be advantageous for an industrial application as any additional processing steps for lignin derivatization would add to the cost of the lignin.

**Figure 4:**
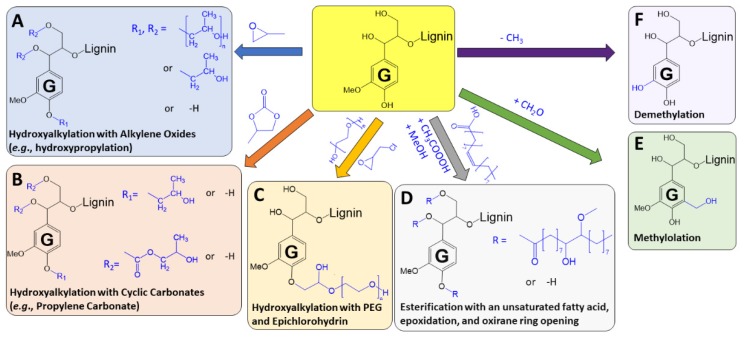
Example derivatization approaches for improving reactivity of a terminal guaiacyl monomer within a lignin polymer with an isocyanate and incorporation into PUs.

A number of chemical functionalization routes have been employed in order to increase a lignin’s reactivity with isocyanates that fall into the general categories of (**1**) hydroxyalkylation and (**2**) demethylation. A number of these functionalization strategies are outlined in Figure 4 for a terminal guaiacyl monomer linked via a β-aryl ether linkage and exhibiting a free phenolic group and a primary and secondary aliphatic hydroxyl group in the side chain region of the lignin. Hydroxyalkylation approaches include introduction of primary (and secondary) alcohols through the addition of hydroxylated alkyl chains at various sites on the lignin polymer. These sites can include primary and secondary alcohols in the lignin side chain region, a free phenolic hydroxyl group, and an unsubstituted aromatic ring ortho to a free phenolic hydroxyl group (i.e., a 3 or 5 position in Figure 2A). It should be considered that many functionalization reactions will exhibit differing reactivities for a primary alcohol, a benzylic alcohol, and a phenolic hydroxyl.

Hydroxyalkylation using alkylene oxides such as ethylene, propylene, and butylene oxide is a well-established approach to increase the accessibility of phenolic hydroxyl groups of lignins and improve their incorporation into polyurethane applications [57,58]. This approach can introduce a hydroxyalkyl group (e.g., a hydroxypropyl) or polyethers of the alkyl oxide (e.g., polypropylene glycol) on any of the accessible hydroxyl groups within the lignin (Figure 4A). It has previously been established that the conversion of a phenolic to a more reactive aliphatic hydroxyl group enables the functionalized polymeric lignin to be used as a polyol component in polyurethane foams [59]. More recent work has demonstrated that hydroxyalkylation can be performed using cyclic carbonates such as propylene carbonate [60,61] as both a solvent and a reactant. These cyclic carbonates have the potential to be more “green” feedstocks as CO_2_ is one of the reactants in cyclic carbonate synthesis. As an example of lignin functionalization, propylene carbonate has been used to hydroxypropylate phenolic hydroxyl groups and is proposed to esterify to non-aromatic alcohols [62] (Figure 4B). This approach has recently been applied to generate polyurethanes from lignosulfonates [63]. Grafting a PEG-chlorohydrin ether onto the phenolic hydroxyl group of lignins has been demonstrated as a route to convert phenolic hydroxyl groups to aliphatic hydroxyls (Figure 4C) [64]. In another approach, an unsaturated fatty acid (e.g., oleic acid) was esterified to lignins and subjected to epoxidation and oxirane ring opening (Figure 4D) to increase the reactivity of lignins and substantially increase the bio-based content of the resulting polyurethane product [65]. Methylolation is another approach to increasing the aliphatic hydroxyl content of lignin (Figure 4E) and involves reaction of lignin with formaldehyde with a base catalyst to introduce methylol groups at an unsubstituted ortho position relative to a free phenolic group [66]. The other main strategy for increasing the aromatic hydroxyl content of lignins is by demethylation of a methoxyl group to yield a catechol moiety (Figure 4F). Strategies for this include acid-catalyzed demethylation using Lewis acids such as 1-dodecanethiol, iodocyclohexane, and hexadecyltributylphosphonium bromide as well as Brønsted-Lowry acids such as hydroiodic and hydrobromic acid [11,67]. 

## 3. Lignin-Based Polyurethanes 

### 3.1. Lignin-Based Rigid Polyurethane Foams 

Rigid polyurethane foams are used in a wide range of applications that include automotive, construction, board stock, sandwich panels, refrigeration appliances, and technical insulation [68,69]. The high demand for rigid PU foams is fueled by a combination of their characteristic properties including low thermal conductivity, good adhesion to a variety of substrates, low density, and high mechanical strength [70]. Polyurethane and polyisocyanurate (high isocyanate index PU) rigid foams are excellent insulators and have one of the highest R-values (resistance to conductive heat) per inch compare to many commercially available insulating products such as cement, fiber glass and polystyrene foam [71]. Additionally, rigid PU foams are exceptionally strong, lightweight, dimensionally stable, and moisture resistant [72]. They can be applied to various substrates and can even be molded into different shapes based on the required applications [69]. Most rigid PU foams can provide structural support due to their high compressive and shear strengths [69]. 

**Figure 5:**
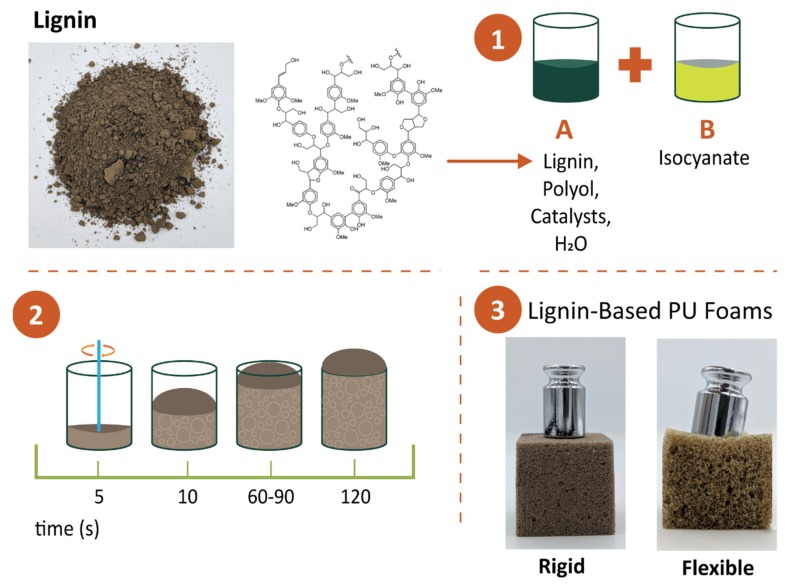
Schematic showing production of rigid and flexible lignin-based polyurethane foam.

Rigid PU foams are prepared by mixing an A-side isocyanate, usually polymeric methylene diphenyl diisocyanate (pMDI), with a B-side polyol blend; a preblended mixture of additives (blowing agents, catalysts, fire retardants, and surfactants) and polyols to create urethane/carbamate linkages [69,73]. The main difference between polyurethanes and polyurethane foams is the use of blowing agent, either chemical or physical (Figure 5). If the blowing agent participates in the reaction, it is classified as a chemical blowing agent (e.g., water) and if the blowing agent does not take part in the reaction, it is classified as a physical blowing agent (e.g., pentane) [74]. Physical blowing agents are converted to the gaseous state due to exothermic reactions during the foam formulation process. Chlorofluorocarbons (CFCs) are the ideal blowing agents due to their exceptional flame retardant and insulation properties, but have been mostly phased out due to their negative environmental impacts that contribute to ozone depletion and global warming [69,75]. While liquid CO_2_ is an inexpensive alternative, most industries use pentane (along with water as chemical/co-blowing agent) due to its reasonable thermal conductivity and cost [69,70]. One of the most important properties of rigid PU foams is their thermal conductivity. Optimal thermal conductivity is obtained when foams have fine, uniform, and high closed cell contents [69]. These properties along with low density are usually achieved when a mixture of physical and chemical blowing agents are used together [69]. 

Unless an amine-based polyol is used, the first reaction in rigid PU foams is between water and an isocyanate [69]. This is an exothermic reaction that leads to the formation of an amine and CO_2_ gas. The amine continues to react with more isocyanate to create polyurea and the CO_2_ is trapped inside the foam cells as a blowing agent [76]. The second reaction is usually between an isocyanate and a polyol. This is another exothermic reaction that creates polyurethane and isocyanate trimerization (i.e., polyisocyanurate) [69]. Each reaction; water/isocyanate, polyol/isocyanate, and polyisocyanurate can be individually catalyzed through the addition of blowing agent, gelation agent, and trimerization catalysts, respectively [69]. 

The key to rigid PU foam formulation is the balance between raw materials, processing parameters, and foam properties. Crucial factors in rigid PU foam formulations are the polyol/isocyanate ratio, the type of isocyanate, and its index [69]. A wide range of polyols can be used to create rigid foam but the main types are sorbitol-, sucrose-, or glycerol-based polyether, amine-based polyols, and aromatic polyester polyols [69,77]. The choice of suitable polyols varies based on the final application, but ultimately depends on their compatibility with the blowing agent. Whichever polyol is chosen, the final polymer must be homogeneous and highly crosslinked in order to create foam with low density, low thermal conductivity, and high compression strength [69]. 

The majority of the isocyanate and polyol used in rigid foam polyurethanes are petrochemically sourced from phosgene and propylene oxide respectively [69]. Fluctuations in the price of oil and gas creates uncertainty in the market price of these raw materials. Diminishing resources [78], rising prices [79], and greenhouse gas emissions associated with fossil-derived feedstocks have resulted in increased efforts to include bio-based and green alternatives in polyurethane synthesis [80,81]. Incorporating bio-based materials into the formulation of polyurethanes will increase the economic and environmental benefits of final polyurethane products [81,82]. 

Vegetable oil and other bio-based alternatives have been explored for the preparation of polyurethanes. But it is now more important than ever to create polyurethanes that are not only sustainable but also cost-effective and functional. Lignin is an attractive polyol for industry, because unlike vegetable oils, it does not compete with food resources [83]. Lignin proves to be a viable substitute for petrochemical based polyols due to its low cost, high abundance, reactive functional groups, and decreased isocyanate consumption when incorporated into polyurethanes [78,84,85].

Although exhibiting much lower reactivity, unmodified lignin can be directly incorporated into PU foam formulations [86]. Higher incorporation of unmodified lignin over 30 wt % has been reported to result in brittle foams with decreased compression strength [24,87,88]. This is due to the poor cellular structure [88,89] of lignin-based rigid PU foam along with the aromatic structure of lignin which increases the ratio of hard segments creating a brittle and friable foams [90,91]. This problem has been addressed through the addition of chain extenders like castor oil [92], polypropylene glycol triol [92], and butanediol [93]. Elongation at break and compression strength are some of the properties that have been reported to increase with the addition of chain extenders due to increased flexibility they impart [90,92,93]. The main challenges in incorporating higher proportions of lignin in polyurethane foams are increasing viscosity [56], [94] and density [95], while decreasing curing/reaction times [70], and in some cases, unpleasant odor usually associated with kraft softwood lignins [96]. While the increased density leads to subsequent increases in compression and Young’s moduli [88,97], the increase in viscosity creates difficulties during formulation and application processes at the industrial scale. As described previously, the main concerns with using lignin as a bio-based polyol are its dark color and low reactivity with isocyanates [98], which can be linked to the high molecular weight of lignin [99,100]. The dark color of lignin will not be a problem for some applications (i.e., insulation where foams are not seen). While, low reactivity of lignin with isocyanate can be addressed by chemical modification, or choosing a more reactive lignin [101]. 

Oxypropylated lignins have been incorporated into rigid PU foams at 5–100 wt % loading with “comparable performance” to control foams [15,70,94,102]. The incorporation of modified lignins into rigid PU foams has been reported to improve biodegradation [103], compressive strength [104,105,106], tensile strength [107], thermal conductivity [108], thermal degradation [109], and anti-flaming properties [110] compared to control foams. In some cases it is also reported to increase density [106], cell size [84], and cell irregularity [88] of the foams.

Although hydroxypropylation produces lignins that are more suitable for PU applications, the use of petrochemically sourced propylene oxide, high pressures, and long reaction times may make these modifications economically prohibitive [111,112]. Multiple studies have depolymerized lignins using NaOH (with and without isopropanol-water solvent, respectively) [113,114] and concluded that the depolymerized lignin had hydroxyl values in the acceptable range for polyurethane foam synthesis (~350 mg KOH/g) [114]. 

Although a number of different lignin types have been incorporated into rigid polyurethane foams such as lignosulfonate, soda, kraft, and enzymatic hydrolysis lignins, the correlation between lignin properties and foam performance are not well-established. Additionally, more detailed studies are needed to determine the impact of lignin incorporation on the price, feasibility, and formulation of rigid PU foam at the industrial scale.

### 3.2. Lignin Based Flexible Polyurethane Foams

**Figure 6:**
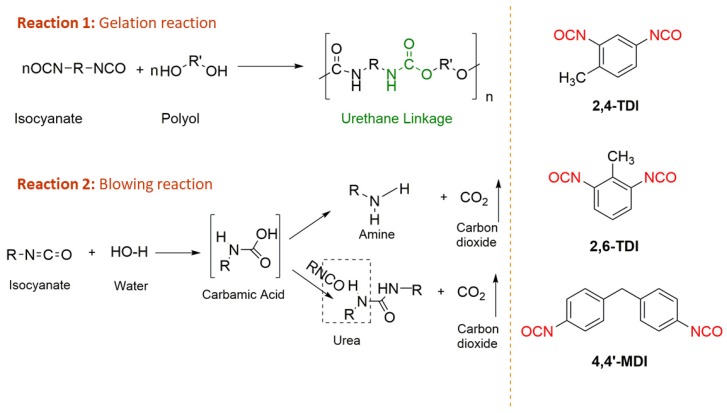
Schematic reaction of isocyanate and water (blowing agent) and the formation of urethane linkage and commonly used isocyanates (TDI and MDI).

Flexible PU foams have been widely utilized in consumer and commercial products including furniture, carpet cushion, transportation, bedding, packaging, textile, and fiber applications [92,115]. Generally, flexible PU foam formulations differ from rigid PU foams by including polyols with lower hydroxyl (OH) values, using water as the sole blowing agent, and using toluene diisocyanate (usually a 80:20 mixture of 2,4- and 2,6-isomers) along with varied catalysts and surfactants [116,117]. There are two primary reactions in the production of flexible foams (Figure 6): the first reaction is between a hydroxyl group within a polyol and an isocyanate group to form a urethane linkage. The second reaction is between an isocyanate and water that not only generates carbon dioxide as the blowing agent, but also extends the polymer chains through the urea linkage providing both covalent and hydrogen bonding sites. The resultant foams contain a high number of open cells that result in improved dimensional stability and the desired mechanical properties required for flexible foams [118]. Additionally, flexible polyurethane foams can be considered as copolymers with hard polyurea blocks connected by long flexible chains through urethane linkages. The hard segments with hydrogen bonding contribute to the firmness of the polymer, while soft segments provide elasticity to the foam. Changing the ratio of hard and soft segments during formulation can yield foams with different properties designed for specific applications.

Lignin incorporated into flexible PU foams can act as both a filler [119] and crosslinking agent (by reacting with an isocyanate) [106]. Previous studies have used either unmodified lignins [95,106] or chemically modified lignins [120,121,122] to partially replace (6–30 wt %) the polyol portion of flexible PU foams. The thermal stability and mechanical properties foams prepared using modified lignins were reported to increase by increasing lignin content [120,121,123]. 

Kraft softwood lignin has been used to replace 10–30% (by weight) of the polyol in flexible foam formulations and reported that the developed foam had viscoelastic behavior similar to the control foams [106]. The foam produced with 100% replacement of the polyol with sodium lignosulfonate lignin was dense, brittle, and mechanically weak and was more like a rigid foam than flexible foam [123]. Overall, the prepared foams using kraft lignins and lignosulfonates were reported to have better compressive strength [95,106], viscoelastic properties [106], and dimensional and thermal stability [95,106,123] relative to the control foams using conventional fossil-derived polyols. Bernardini et al. [120] found that oxypropylated soda lignin was more soluble in the co-polyols (glycerol and polyethylene glycol-400) and exhibited better miscibility with chain extenders (polypropylene glycol triol and castor oil). The foam obtained from the modified soda [120] or kraft [92] lignin had high open cell content and thermal and mechanical properties making them suitable for packaging and automotive applications.

Wang et al. [121] grafted long polyethylene glycol (PEG 2000) chains onto an alkali lignin and reported that the introduction of PEG-2000 improved the compatibility of the PEG grafted-lignin and improved the flexibility of developed foams compared to the foam prepared by using the unmodified alkali lignin. Also, reported that the flexible PU foams made by replacing 50% of the polyol with the modified lignins, exhibited elastic recovery higher than 93% with desirable resilient performance [121]. The compression modulus and the compression force deflection values of the foams also increased significantly with the increase in modified lignin content [121].

### 3.3. Lignin Based Polyurethane Elastomers

Polyurethane elastomers have been widely used in variety of applications including flooring (e.g., indoor basketball and volleyball court flooring), automotive interiors [124], surfboards, footwear, and home and kitchen appliances [2], and are predicted to share approximately 10% of total polyurethane production in the world by 2020 [2]. Lignin has the potential to be used in polyurethane elastomer synthesis; however, the high glass transition temperature (and consequently low flexibility) of lignin has restricted its application in elastomers.

Lignin has been used as a filler in polyurethane elastomers and it is known to enhance the mechanical properties of the elastomer [125]. The interaction between filler and matrix can strongly affect the elastomer’s Young’s modulus. Different lignins interact differently with the elastomer matrix due to differences in their surface energies and their adhesions to the matrix. Lignins with a higher equilibrium work of adhesion are more likely to contribute to mechanical strength of the elastomer, thus increasing the tensile modulus [125]. 

A few types of lignins such as a kraft softwood lignin, a sulfite hardwood lignin, a soda wheat straw lignin [126], and a lignin-derived aromatic dimers [127] have been investigated for synthesis of PU elastomers. It was reported that the elastomers made with soda wheat straw lignin had higher storage modulus than other lignin [127]. Lignin acts as the rigid component in polyurethane elastomer matrix, and normally has higher glass transition temperature and Young’s modulus than the matrix. It is reported that by increasing the amount of lignin incorporation into the PU matrix, the glass transition temperature and mechanical properties of the matrix such as Young’s modulus and storage modulus increased [107,126]. Lang et al. [126] reported that incorporating up to 60% (by weight) lignin into PU elastomers increased the storage modulus up to 6-fold relative to the control sample without lignin. Li et al. [107] studied the effect of lignin molecular size on elastomer properties and reported that replacing up to 40% of the polyol with a low molecular weight lignin (600 g·mol^-1^) resulted in a more homogenous elastomer with higher mechanical strength.

### 3.4. Lignin-Based Polyurethane Coatings 

Polyurethane coatings are currently used for wood, metal, plastic, leather, and textile applications. The automotive and transportation industry are the leading consumer of PU coatings due to their high dielectric strength and high chemical, heat, and weather resistance properties [128]. 

Different sources of lignins including wheat straw [129], organosolv [24], and kraft lignin [130] have been used to partially (40 to 80% by weight) replace polyol in PU resin formulations used for coating applications. To increase the incorporation of lignin into coating formulations solvent fractionation and chemical functionalization have been used. In two studies of solvent fractionation, 2-methyl tetrahydrofuran (a potentially bio-based solvent ) [131] and 1,4-butandiol [132] were used to separate soluble segments of lignin from insoluble lignin parts. As a result, they obtained more homogeneous lignin fractions with lower molecular weights, which were more monodisperse, and completely soluble in the solvent used for extraction. 

Liu et al. [22] prepared a waterborne PU coating using aminated lignins derived from alkaline pulping as a bio-based diisocyanate replacement. The aminated-lignins were used to prepare PU prepolymers by mixing the aminated-lignin with toluene diisocyanate and poly(propylene) glycol. They reported that by increasing the amount of aminated lignin in resin formulation the mechanical properties and weathering performance of formulated coating improved significantly. Mozheiko et al. [133] used a hydroxypropylated lignosulfonate and showed that the coatings prepared with hydroxypropylated lignin had higher solvent and weathering resistance than the coating prepared with commercial petroleum-based polyols. Zhang and Huang [134] mixed a nitro-lignin, synthesized by reacting alkali lignin with nitrosonitric acid and acetic anhydride, with castor oil to prepare PU system with different ratios of NCO/OH. The prepared coatings with inclusion of nitrolignin showed superior water and UV resistance and higher dimensional stability compared with uncoated cellulosic film [135]. 

### 3.5. Lignin Based Polyurethane Adhesives

Common adhesives such as urea, melamine, and phenol-formaldehyde are highly sensitive to hydrolysis and produce environmental and health problems due to volatilization of formaldehyde over the lifetime of product [8]. Compared to these commonly used adhesives, polyurethane (PU) adhesives have the demonstrated advantages of improved strength, lower curing temperatures, less press time, and no formaldehyde emissions [136]. PU adhesives can possess some of the best performance characteristics of all adhesives, and they are particularly important in the production of engineered wood products (e.g., cross-laminated timber) as a binder [137]. They are colorless and moderately flexible and can be used for bonding wood, metal, glass, plastic, rubber, ceramic, sand, and textile fiber [8]. Some drawbacks associated with PU adhesives include high penetration of the adhesive into the wood, low resistance to delamination, and unsatisfactory gap filling properties. Although addition of rigid fillers such as calcium carbonate can overcome these issues by increasing the viscosity, it does not resolve the gap-filling problem [138]. Lignin is a promising solution to address these challenges. It has been reported that addition of lignin into PU adhesive formulations improved the delamination resistance, cohesive failure, and improved the gap filling properties [138]. Specifically, this study showed that the glue line delamination was decreased by 45.5% in a sample containing 20% (by weight) Alcell lignin (an organosolv lignin). 

Lignin incorporation into the polyurethane matrix as a polyol replacement can increase the aromatic content of the final polymer. It has been found that at high temperatures, lignin remains moderately stable and acts as a thermosetting material due to its aromatic and crosslinked structure [139,140]. Consequently, it is expected that incorporation of lignin would increase the glass transition temperature and thermal stability of the adhesive [141,142]. 

Composition, chain length, and number of hard and soft segments are the parameters that strongly affects the glass transition temperature, thermal decomposition, and mechanical strength of lignin-based polyurethanes [9,142]. Chahar et al. [141] reported that the shear strength decreased as the chain length of the polyethylene glycol (PEG) was increased and the optimum properties were obtained for a PU containing 50% *w/v* lignin in polyol (PEG with molecular weight of 200). Additionally, according to their result, the initial decomposition temperature increased from 270 to 285 °C as lignin content increased to 50% *w/v* of polyol [141].

The mechanical properties of polyurethanes are mainly determined by crosslinking density, which is dependent on the stoichiometry and functionality of its reactants [142]. Although lignin replaces polyol in the PU structure (the soft segment of the PU), it usually serves as the hard segment due to its aromatic structure, and consequently its addition increases the mechanical strength [142,143]. 

Lima García et al. [138] tested the lap shear strength of polyurethane adhesives using different lignin types (an organosolv lignin and two high-purity kraft lignins) as fillers and evaluated the effect of different dispersion methods (high speed mixer and three-roll mill) on the performance of the adhesives. They showed that the adhesive prepared from Alcell lignin (as a filler) had a higher shear strength compared to kraft lignins. They found that addition of 20 wt % lignin into the resin formulation increased stress at break by nearly 55%. Overall, the results indicated that all the resins containing lignin had cohesive failure, but comparable gap filling performance to the reference filler (fumed silica). 

Another way to improve lignin reactivity toward isocyanate is modifying lignin through functionalization as discussed in the previous section. Bonini et al. [144] reported that modified lignin (lignin recovered by NaOH extraction of steam-exploded straw) exhibited higher reactivity toward isocyanate. Hsu and Glasser [143] copolymerized lignin with maleic anhydride and then hydroxyalkylated lignin using propylene oxide to make a more reactive polyol for both adhesive and coating applications. They identified that the source of the isocyanate, the solvent, and lignin source were the primary factors affecting shear strength of the adhesive product. Adhesives formulated with 4,4’-diphenylmethane-diisocyanate (MDI) and dimethylformamide (DMF) showed the highest shear strength. In other work, the same group used either organic solvent or emulsified formulations and investigated the effects of lignin source, crosslinking agents (isocyanate and melamine), and lignin derivatives on adhesion performance [145]. In the emulsion system, both crosslinking reagents showed approximately the same shear strength. They found that adhesive performance was related to the compatibility and solubility of components in the emulsion system, while for the solvent-based system the performance was primarily affected by the molecular weight of the lignin derivatives. Their results showed that all lignin-based adhesives demonstrated performance that was equal to or better than that of the reference urea-formaldehyde adhesive [145]. In another study, they investigated the potential use of functionalized lignin derivatives as a binder [146]. Southern yellow pine particle board was prepared using mixture of polymeric MDI with different amount and types of lignins. The modulus of rupture results showed that alkali-extracted lignin from steam exploded aspen chips, had satisfactory performance in binding the particle board. This was hypothesized to be due to the higher reactivity of this lignin with isocyanate and its low molecular weight. It was observed that by reacting 30 (wt %) hydroxypropylated lignin with isocyanate, the strength of the binder was greater than the pure isocyanate adhesive (polymeric MDI). Also, addition of lignin up to half of the total weight of isocyanate did not result in losses in the mechanical strength [146].

Lignin generally improves mechanical strength and thermal properties of blend, copolymers, and composite materials, due to its aromatic structure. The amount and chemical structure of the lignin will affect the properties of the final adhesive. It has been reported that the incorporation of lignin in polyurethane matrix could greatly enhance the thermal stability [141], delamination [138], and abrasion resistance [147]. However, using higher amounts of lignin more than 60 wt %. in total [145] reported to increase brittleness [142] and cause phase separation [9], which would result in poor stress transfer and ultimately weakening the mechanical properties of the final products [9,126,141]. 

## 4. Conclusions

The markets for polyurethanes and polyols have shown strong, sustained growth in recent years and provide an opportunity for integration of new bio-based feedstocks for improved sustainability. Lignin has the potential to be utilized as renewable, bio-based replacements for polyols currently derived from petroleum and natural gas in the synthesis of diverse polyurethane products. While substantial work has been directed at the use of wood lignins derived from chemical pulping processes, biorefining processes using non-wood feedstocks can yield lignins with significantly different properties. Additionally, novel approaches for fractionation and functionalization can yield lignins with properties tailored to the target application.

## Figures and Tables

**Table 1:** Potential challenges for integration of lignins into polyurethane (PU) products and possible mitigation approaches.

Lignin Property	Impact on PU Synthesis or Performance	Potential Solution		
Sulfur content	Odor problems in final product, yellowing in coating applications	Selection of appropriate lignin source		
Low solubility	Poor incorporation into polymer matrix and low interaction with the co-reactant (NCO)		Depolymerization or fractionation of the lignin	Functionalization of the lignin
Low reactivity	Poor incorporation into polymer matrix			
High *T_g_*	Brittleness			
High polydispersity	Inconsistent performance, differing reactivities and solubilities			
Dark color	Limited utility in some coating applications where light color is required			
Ultraviolet (UV) instability	Product degradation in outdoor applications	Ensuring the reaction between isocyanate and lignin phenolic hydroxyl groups; Addition of UV stabilizers		
